# Construction of eco-quality dual-suitable distribution areas for *Dictamnus dasycarpus* Turcz.: integrated analysis of MaxEnt model and multidimensional indicators

**DOI:** 10.3389/fpls.2025.1591921

**Published:** 2025-07-16

**Authors:** Boqian Jiang, Dan Wang, Qiuju Ye, Jiayi Luo, Bingqian Jin, Haibo Yin

**Affiliations:** School of Pharmacy, Liaoning University of Traditional Chinese Medicine, Dalian, LiaoNing, China

**Keywords:** *Dictamnus dasycarpus* Turcz., Maximum Entropy model, Geographic Information System, ecological suitability, quality zoning, Geodetector

## Abstract

**Introduction:**

*Dictamnus dasycarpus* Turcz., a critical traditional medicinal plant in Northeast China, faces challenges of habitat degradation and unstable quality in cultivated populations.

**Methods:**

This study systematically analyzed the key environmental drivers of its distribution and quality formation in Liaoning Province through an integrative framework combining the Maximum Entropy model (MaxEnt), High-Performance Liquid Chromatography (HPLC), and geodetector analysis.

**Results:**

July precipitation (Prec7), temperature seasonality (Bio4), May solar radiation (Srad5), March maximum temperature (Tmax3), and March minimum temperature (Tmin3) were core variables influencing distribution patterns. The quality of cultivated populations was primarily regulated by February mean temperature (Tmean2) and May precipitation (Prec5), while that of wild populations were predominantly affected by January mean temperature (Tmean1). By overlaying ecological suitability zones, quality partitions, and existing planting areas, Chaoyang, Huludao, Jinzhou, Liaoyang, and Dandong were identified as ideal regions combining ecological adaptability and quality advantages.

**Discussion:**

The study revealed that precipitation and temperature are key factors affecting both distribution and quality. Geodetector analysis confirmed significant interactions among environmental variables influencing both distribution and quality. The multi-model framework established in this study provides a scientific basis for precision cultivation zoning and wild resource conservation of medicinal plants. The identified high-quality planting regions can promote the sustainable development of the *D. dasycarpus* industry, and the methodological approach provides a reference for similar studies.

## 1 Introduction

Traditional Chinese Medicine (TCM) encompasses a class of therapeutic agents applied under the guidance of TCM theory for the prevention, treatment and diagnosis of diseases, as well as for rehabilitation and health care (Liu et al., 2016). TCM has been continuously developed and utilized by Chinese people over thousands of years as a fundamental approach to prevent and treat diseases (Guo et al., 2012). Nevertheless, the complexity and diversity of TCM components present significant challenges to quality control (Xing et al., 2023). The quality of TCM can be influenced by multiple factors and is not only generated by the expression of intrinsic genes that govern the metabolic pathways of active ingredients (Yang et al., 2022), but also by the ecological environment (Camina et al., 2023). In particular, the ecological environment plays a crucial role in influencing the growth and development of medicinal plants and the production of secondary metabolites (Neugart et al., 2018; Sha et al., 2023; Cheng et al., 2024). However, excessive stress can cause adverse effects, potentially leading to the death of medicinal plants (Prasch and Sonnewald, 2013; Suzuki et al., 2016; Srivastava et al., 2009; Pant et al., 2021). Consequently, investigating the relationship between medicinal plants and ecological factors is indispensable if we are to achieve comprehensive levels of quality control in TCM. By understanding how ecological factors interact with medicinal plants, we can better regulate and optimize cultivation conditions to ensure the stable quality of TCM materials.

*Dictamnus dasycarpus* Turcz. (hereafter *D. dasycarpus*), a member of the Rutaceae family and the genus *Dictamnus* L., is a perennial herb characterized by its pungent odor. This herb serves as the base plant for Dictamni Cortex, a well-known TCM in the northeast of China (Lv et al., 2015; Bai et al., 2011). The root bark of *D. dasycarpus*, which is the sole authentic source of Dictamni Cortex, possesses significant therapeutic properties, including the ability to clear heat, dry dampness, dispel wind, and detoxify (Qin et al., 2021). Modern pharmacological studies have revealed that *D. dasycarpus* exerts multiple beneficial effects, with remarkable efficacy particularly evident in its anti-inflammatory actions (Choi et al., 2016; Gao et al., 2021; Jung et al., 2024). For instance, both Choi et al. (2019) and Kim et al. (2013) provided compelling evidence of the potent anti-inflammatory properties of this herb. In addition to its anti-inflammatory effects, *D. dasycarpus* also exhibits notable antioxidant potential. In a previous study, Guo et al. (2016) reported significant antioxidant effects, contributing to the mitigation of oxidative stress within the body. *D. dasycarpus* has also yielded promising results in terms of cancer research. Park et al. (2015); Zhang et al. (2022) and Zuo et al. (2024) documented the ability of *D. dasycarpus* to inhibit the growth of certain cancer cell lines. Furthermore, *D. dasycarpus* possesses antihyperglycemic and anti-allergic properties (Lv et al., 2015). In addition to its pharmacological effects, *D. dasycarpus* holds an abundance of active compounds, including quinoline alkaloids, limonoids, sesquiterpenes, coumarins, flavonoids and steroids (Gao et al., 2022). Of these chemical constituents, quinoline alkaloids and limonoids play a pivotal role in terms of medicinal value (Chen et al., 2020). Overall, these diverse pharmacological effects and compounds highlight the significant value of *D. dasycarpus* in the field of medicine and warrant further exploration and utilization.

*D. dasycarpus* is widely distributed in Liaoning Province, and is characterized by abundant resources and strong adaptability (Bai et al., 2011; Zhi et al., 2022). This herb commonly grows on sunny hilly slopes, forest edges, low shrublands, grasslands, sparse forests and limestone mountains (Jia et al., 2022). *D. dasycarpus* prefers a humid and warm climate, and is tolerant to light cold and drought conditions but intolerant of waterlogging or flooding. *D. dasycarpus* thrives best in fertile, loose and well-drained sandy loam soils on flat or gentle slopes. However, *D. dasycarpus* cannot grow readily in low-lying areas prone to waterlogging, saline-alkali soils, or heavy clay soils (Wang et al., 2023). Despite its medical significance, wild *D. dasycarpus* resources have been severely depleted due to urbanization and agricultural expansion, while cultivated varieties often exhibit inconsistent quality (Wang et al., 2023; Zhi et al., 2022), necessitating systematic research on its ecological suitability and quality formation mechanisms.

Field investigations have revealed that commercial Dictamni Cortex in Liaoning Province is predominantly sourced from cultivated plants. However, due to the relatively long growth cycle, of this plant, large-scale cultivation has yet to be fully achieved. Approximately 25% of the annual supply continues to originate from wild sources. This variability in origin results in inconsistent product quality (Huang and Guo, 2007), thus creating a significant hinderance on the industrial development of *D. dasycarpus*. Consequently, to better understand and address these challenges related to quality and industrial development, it is imperative that we gain a comprehensive understanding of the distribution of *D. dasycarpus* in ecologically suitable and high-quality areas within Liaoning Province. Regrettably, there is currently a paucity of systematic reports on this topic, making it challenging for relevant stakeholders to formulate effective strategies for cultivation and industrial promotion.

Species Distribution Models (SDMs) have emerged as critical tools for predicting plant community distributions through species-environment relationship quantification (Qian et al., 2024; Zhang et al., 2016). These models evaluate habitat suitability via ecological indicator systems, enabling comprehensive assessment of species’ environmental requirements and distribution patterns under varying ecological conditions (Borthakur et al., 2018; Yang et al., 2013).Widely adopted platforms including Climate Explorer (CLIMEX) (Sutherst and Maywald, 1985), Biogeographical Mapping and modelling (BIOMAPPER) (Hirzel and Guisan, 2002), and Maximum Entropy (MaxEnt) (Phillips et al., 2006) each demonstrate distinct advantages. Among these, however, the MaxEnt algorithm has gained particular prominence due to its computational efficiency and robust performance with sparse datasets (Marini et al., 2010; Hengl et al., 2009). These characteristics have established MaxEnt as a preferred modeling approach for global-scale habitat suitability predictions in contemporary ecology.

Spatial differentiation is a fundamental research topic in geography (Wang and Xu, 2017). Geodetector is a comprehensive tool that is used to detect and analyze spatial differentiation, incorporating a suite of statistical methods that can reveal the driving forces underlying such differentiation (Liang and Xu, 2023; Wang and Xu, 2017). Geodetector features successive detection ability for factors, interactions, risk and ecology (Zhang et al., 2021). During the growth and development of medicinal plants, the ecological environment plays a crucial role in determining the quality of medicinal materials (Ma W. et al., 2021). Wild and cultivated medicinal plants experience different environmental conditions during their period of growth, thus leaving plants vulnerable to varying impacts from environmental variables. Therefore, when investigating the ecological suitability zones of TCM and the accumulation of key medicinal components, it is essential to consider not only the influence of dominant environmental variables but also the combined effects of these variables (Yu et al., 2023). Geodetector has been widely applied in analyzing the spatial differentiation characteristics of medicinal plant indicators (Wu et al., 2020; Wang and Hu, 2012; Lin et al., 2022) to elucidate the specific effects of environmental variables on the growth and quality of medicinal plants (Shi et al., 2017; Li et al., 2019; Xi et al., 2021).

Over recent years, the increasing demand for Dictamni Cortex has led to a decline in wild *D. dasycarpus* reserves and a steady rise in market prices. To protect wild resources and cultivate high-quality *D. dasycarpus*, it is crucial that we identify suitable areas and sthe patial distribution patterns of *D. dasycarpus* in Liaoning Province. In the present study, we employed the Maxent model combined with geographic information system techniques to analyze the distribution of *D. dasycarpus* in Liaoning Province, considering climate, topography and soil factors. Using this approach, we aimed to identify the dominant environmental factors influencing the potential distribution of *D. dasycarpus* (Xu et al., 2023). We also used HPLC to determine the predominant active components of Dictamni Cortex, establish a regression model between environmental variables and active components, and create a quality partition map for *D. dasycarpus* using ArcGIS software (Xu et al., 2021). In addition, we used Geodetector to analyze the environmental variables affecting the ecological suitability and quality of *D. dasycarpus*. Our study innovatively integrates the MaxEnt model, HPLC, and geographical detector to establish a comprehensive research framework capable of predicting species’ ecological suitability and quality distribution, elucidating the mechanisms driving their spatial patterns, and providing a reference for related studies on other species. The results visually explained the distribution of high-quality *D. dasycarpus* in Liaoning Province, predicted potential planting areas, and provided a scientific basis for quality control, wild resource protection, and the selection of optimal planting areas both currently and in the future.

## 2 Materials and methods

### 2.1 Sample and data collection

Liaoning Province, situated in the southern sector of Northeast China (41°43′30″N to 38°43′00″N, 118°53′00″E to 125°46′01″E), constitutes a unique geopolitical entity within the Northeast region as the sole provincial administrative unit possessing dual coastal and border advantages. The province encompasses a total territorial area of 148,600 km², complemented by a maritime jurisdiction spanning 41,300 km² within the Bohai and Yellow Sea basins.The provincial terrain exhibits a gradual southward descent, with elevation decreasing from the eastern and western peripheries toward the central lowlands, forming a tiered geomorphological structure characterized by mountainous-hilly landscapes in the east/west and alluvial plains in the central corridor (Wang et al., 2022). Under a temperate monsoon climatic regime, the province experiences pronounced seasonality, featuring cold winters, hot summers, and a synoptic pattern of coinciding precipitation and thermal maxima.

**Figure 1:**
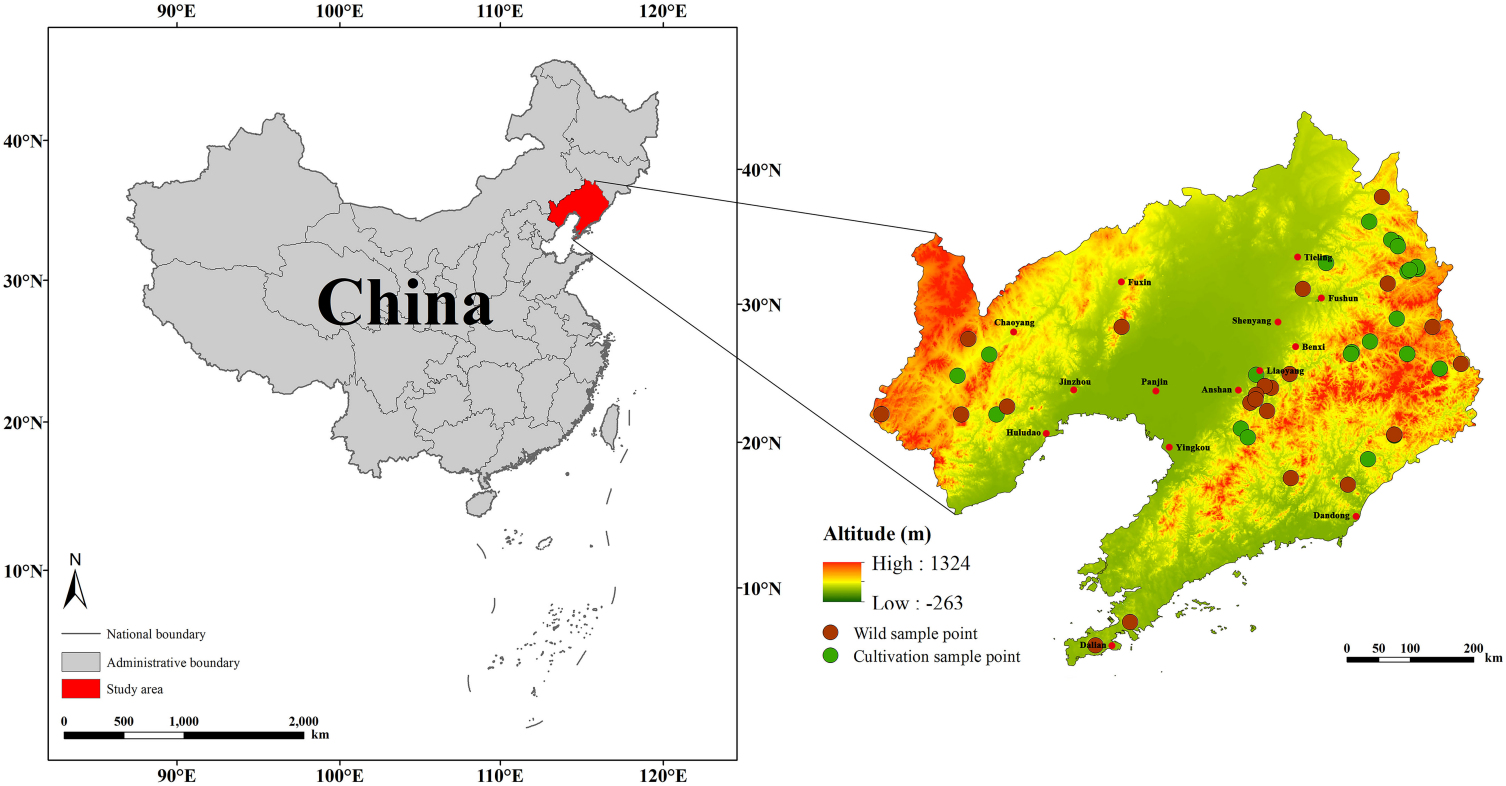
Sampling points for *D. dasycarpus* within the study area.

The *D. dasycarpus* samples used in this study were collected between August 2023 and September 2024 across Liaoning Province. A total of 25 batches of cultivated samples and 24 batches of wild samples were gathered, with each batch containing no fewer than five plants. Plant samples were identified as *D. dasycarpus* by Professor Yin Haibo, Director of the TCM Resources Teaching and Research Section at Liaoning University of Traditional Chinese Medicine (**Figure 1**; **Supplementary Table S1**). After collection, the samples were washed to remove sediment, and the wood core was extracted and stored naturally in the shade. Additionally, we obtained distribution records of *D. dasycarpus* in Liaoning Province from multiple sources: the China Virtual Herbaria (www.cvh.ac.cn), the Global Biodiversity Information Facility (https://www.gbif.org/), and the results of the Fourth National Survey of TCM Resources. The map of Liaoning Province used in this study was downloaded from the National Fundamental Geographic Information System Network (http://nfgis.nsdi.gov.cn/). During the course of data processing, we used the ENMTools R package (GitHub: danlwarren/ENMTools) to filter the distribution records meticulously. Points characterized by ambiguous geographic information, duplicates and outliers were all rigorously excluded. Finally, we retained 237 distribution points of *D. dasycarpu* for subsequent research.

### 2.2 Environmental variables

A total of 106 environmental variables (**Supplementary Table S2**) were selected based on the growth and biological characteristics of *D. dasycarpus*. These variables were chosen from the TCM Resources Spatial Information Grid Database (http://www.tcmresources.com/), including vegetation types, Kira index, topography and soil data. In addition, climate data, including precipitation, temperature, radiation intensity and water vapor pressure were obtained from WorldClim2.1 (www.worldclim.org) at a spatial resolution of 30 arc seconds. DEM (digital elevation model) data were acquired from the Geospatial Data Cloud Platform of the Computer Network Information Center, Chinese Academy of Sciences (http://www.gscloud.cn). We utilized these environmental variables to investigate the ecological suitability and quality distribution of *D. dasycarpus*.

### 2.3 Generation of a species distribution model

The MaxEnt model, grounded in the maximum entropy principle, predicts species distributions by integrating records with environmental variables to generate the most uniform probability distribution constrained by observed data (Tang et al., 2021; Liu et al., 2021). Key procedures include data standardization, nonlinear feature transformations, training-test set partitioning, and AUC (Area Under the Curve) validation (Halvorsen et al., 2015). The model outputs probability-based suitability maps, predictor importance metrics, and single-variable response curves, while employing regularization to prevent overfitting (Feng et al., 2019).

In this study, the maximum number of iterations was set to 10^5^ and the convergence threshold was set to 0.0005, with 15% of the distribution points designated as the test set and 85% as the training set. The accuracy of the model was evaluated using the area under the ROC (receiver operating characteristic) curve (Vanagas, 2004; Parodi et al., 2022; Wang et al., 2019; Ma D. et al., 2021). The Jackknife method was employed to assess the importance of each ecological variable, and response curves were used to evaluate the suitability range of environmental variables (Li et al., 2024). Other parameters were set to default values.

To avoid overfitting caused by multicollinearity and autocorrelation among environmental variables (Burgos et al., 2020), a two-step selection process was implemented for environmental variables. First, 106 environmental variables were analyzed using the MaxEnt model, and those with zero contribution were eliminated iteratively until no such variables remained. Second, correlation analysis was conducted on the remaining variables, and those with high correlation (|r| ≥ 0.8) were excluded. For highly correlated factors, only those with greater ecological significance were retained. Finally, we performed 10 repeated modeling operations to ensure robustness.

### 2.4 Analysis of the ecological suitability of *D. dasycarpus*

Based on the MaxEnt model results, we next used ArcGIS 10.7 software to visually output the findings and applied the natural breakpoint classification method (Jenks) to divide the results into four suitability categories: unsuitable (0–0.2), low suitability (0.2–0.4), moderate suitability (0.4–0.6), and high suitability (0.6–1) (Yan et al., 2021; Zhao et al., 2021; Wang et al., 2024a). Using this classification principle, we delineated the ecological suitability zones of *D. dasycarpus* under the current climate change scenario.

### 2.5 Quality zoning analysis of *D. dasycarpus*

The medicinal value of *D. dasycarpus* is primarily attributed to its diverse bioactive constituents, with quinoline alkaloids and limonoids identified as the key bioactive compounds (Gao et al., 2022; Chen et al., 2020). These components exhibit multifaceted pharmacological activities and serve as critical markers for quality assessment of *D. dasycarpus*.

Quality zoning analysis utilized a method that was adapted from Cao et al. (2018) and optimized for the detection of limonin, dictamnine, obacunone, and fraxinellone in 49 batches of *D. dasycarpus* samples. To determine the specific content, we first weighed 1.0 g of sample powder and placed this into a 100 mL round-bottom flask. Next, we added 25 mL of 100% methanol and performed extraction by refluxing in a water bath for 60 minutes. After cooling, any weight loss was made up with 100% methanol; the resulting sample was then shaken well and filtered through a 0.22 μm microporous membrane to generate a test solution. Next, we accurately weighed an appropriate amount of each standard reference substance and dissolved this in 100% methanol to prepare standard solutions. The conditions used for chromatography were as follows: column: Waters Symmetry^®^ C18 (250 × 4.6 mm, 5 μm); mobile phase: acetonitrile (A) and pure water (B); gradient elution program: (0~8min, 45%A~55%A; 8~20min, 55%A); detection wavelength: 228 nm; flow rate: 0.8 mL/min; column temperature: 35°C; injection volume: 5 μL. Under these chromatographic conditions, the chromatographic peaks of each component are well separated.

To investigate the large number of environmental variables used in this study, we first applied preliminary screening. Grey relational analysis (Suo et al., 2024) was first employed to analyze the relationship between the content of chemical components and the extracted values of environmental variables. Based on the variables ranked by Grey Relational Analysis, we analyzed their correlation with chemical components using SPSS version 27.0. (IBM, Chicago, IL, USA). When the correlation coefficient between two environmental variables reached or exceeded 0.8, one of them was discarded (Kumar et al., 2014). Finally, stepwise regression was used to establish the relationship model between chemical components and environmental variables. This model was then imported into ArcGIS software to create distribution maps for single components and the comprehensive quality of *D. dasycarpus*.

### 2.6 Geodetector analysis of significant environmental variables

The Geodetector is a specialized statistical tool designed to identify and interpret spatial heterogeneity (). Its core function lies in quantifying the explanatory power of environmental factors on spatial distribution patterns (quantified by the q-statistic). The unique strengths of the Geodetector include: precise quantification of dominant drivers underlying spatial divergence, effective analysis of variable interactions, compatibility with both continuous and categorical data, and methodological complementarity with ecological models such as MaxEnt. These features collectively establish it as an optimal tool for elucidating the complex environmental influences on the distribution and quality of medicinal plants.

Based on ecological suitability and comprehensive quality distribution maps, we next performed factor analysis and interaction analysis using the GD package in R (Song et al., 2020; Wang et al., 2010, 2016). Spatially differentiated environmental variables that exerted significant influence on the ecological suitability and quality of medicinal materials (*p* < 0.05) were then identified. The contribution rates of the leading environmental variables to the ecological suitability and quality of medicinal materials, as well as the combined effects of other variables, were also determined.

## 3 Results

### 3.1 Evaluation of the accuracy of the MaxEnt model and the identification of important environmental variables

**Figure 2:**
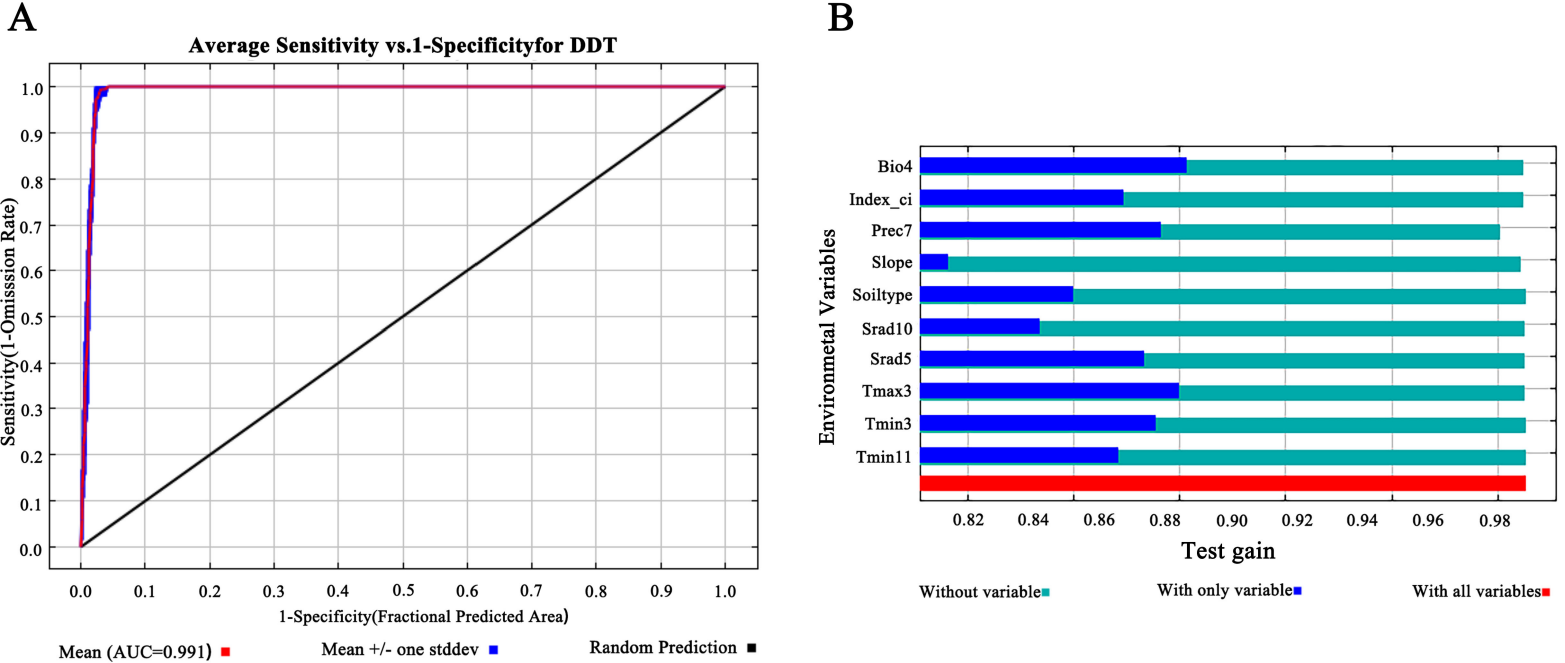
Model accuracy evaluation and the importance of the variables for MaxEnt. **(A)** ROC analysis of Maxent model for predicting the distribution of *D. dasycarpus*. **(B)** Jackknife test results for ecological factors on *D. dasycarpus*.

First, the ROC curve of the MaxEnt model was used to evaluate the accuracy of prediction. We used AUC values as a quantitative measure of model performance; AUC values of 0.5–0.6 indicated failed predictions, 0.6–0.7 indicated poor performance, 0.7–0.8 indicated moderate performance, 0.8–0.9 indicated good performance, and > 0.9 indicated excellent performance (Zheng et al., 2022; Swets, 1988). High AUC values reflected the reliability of the model to predict species distribution (Zhang et al., 2023). The mean AUC of the ROC curve generated by the MaxEnt model after 10 runs was 0.991 (**Figure 2A**), indicating high model accuracy and reliable prediction results for classifying the ecological suitability of *D. dasycarpus* in Liaoning Province.

**Figure 3:**
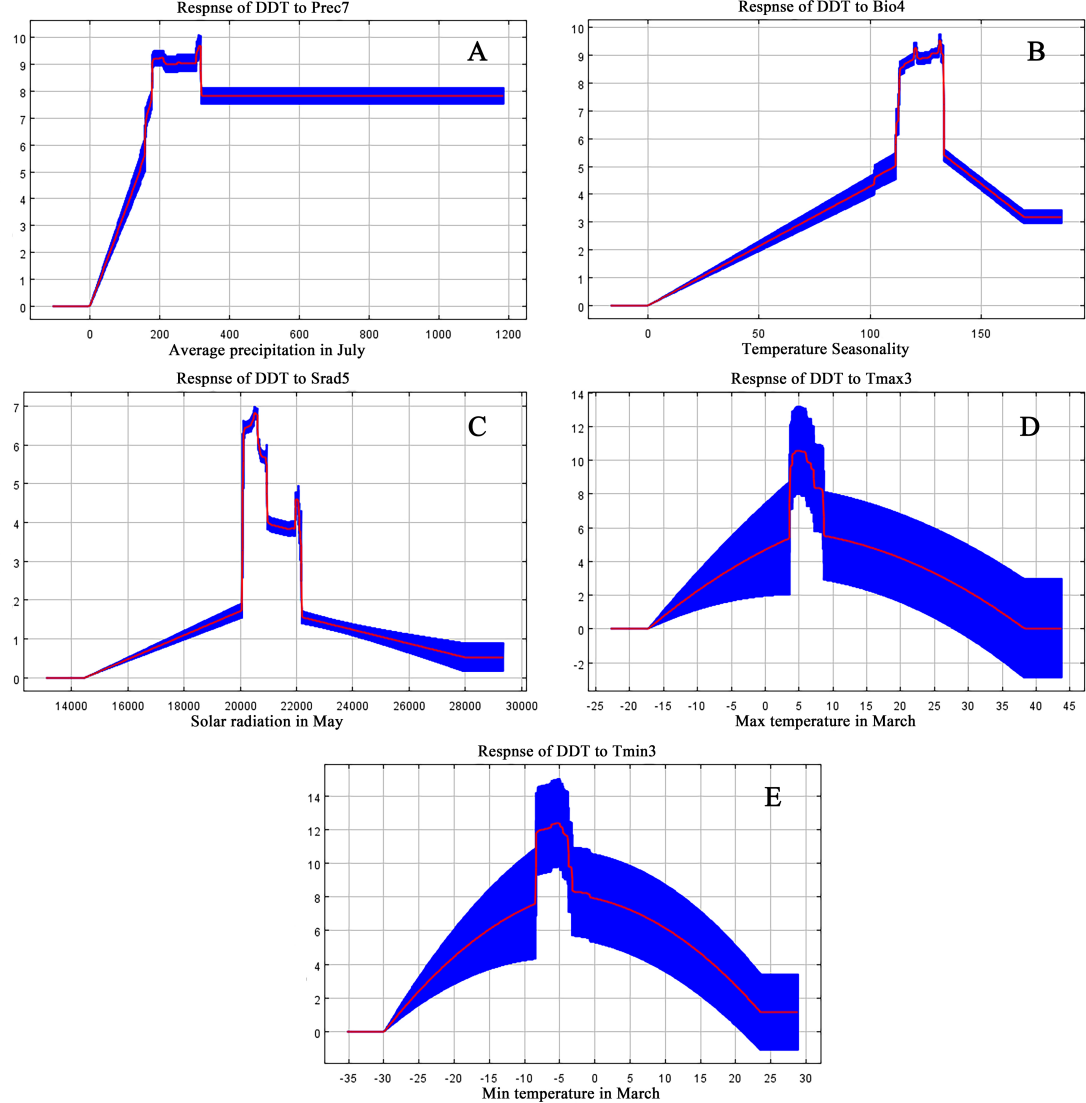
Response curve of *D*. *dasycarpu* existence probability to the dominant environmental variables: **(A)** Average precipitation in July. **(B)** Temperature Seasonality. **(C)** Solar radiation in May. **(D)** Maximum temperature in March. **(E)** Minimum temperature in March.

According to results derived from the MaxEnt model, we identified a total of 10 main ecological factors with non-zero contribution rates (**Table 1**). Of these, precipitation in July (Prec7), temperature seasonality (Bio4), solar radiation in May (Srad5), maximum temperature in March (Tmax3), and minimum temperature in March (Tmin3) cumulatively contributed 95% to the model, thus indicating their dominance in influencing the ecological distribution of *D. dasycarpus*. The Jackknife test further revealed that Bio4 provided the highest regularization gain when used as a single variable, followed by Tmax3 and Prec7 (**Figure 2B**), thus suggesting the critical role of these factors in model performance. Based on response curves, we determined the thresholds for several key environmental variables: precipitation in July (Prec7) ranged from 180 mm to 360 mm, temperature seasonality (Bio4) ranged from 110 to 140, solar radiation in May (Srad5) ranged from 20,000 kJ·m^-2^·d^-1^ to 22,000 kJ·m^-2^·d^-1^, maximum temperature in March (Tmax3) ranged from 4°C to 7°C, and minimum temperature in March (Tmin3) ranged from -8°C to -4°C (**Figure 3**).

**Table 1:** The contribution rate and importance rate of environmental variables.

Environment variables	Percent contribution (%)	Permutation importance (%)
Prec7	47.5	79.3
Bio4	31.9	4.3
Srad5	6.4	1.0
Tmax3	5.1	0.4
Tmin3	4.1	0.6
index_ci	1.8	8.5
Srad10	1.8	1.6
Slope	1.2	3.7
Tmin11	0.2	0.3
SoilType	0.6	0.2

These results demonstrated that precipitation and temperature were the primary environmental variables affecting the distribution of *D. dasycarpus*. Finally, combining the contribution rate table and correlation analysis, we selected Prec7, Bio4, Srad5 and Tmax3 for further analysis.

### 3.2 Prediction of the most suitable ecological areas of *D. dasycarpus* in Liaoning province

**Figure 4:**
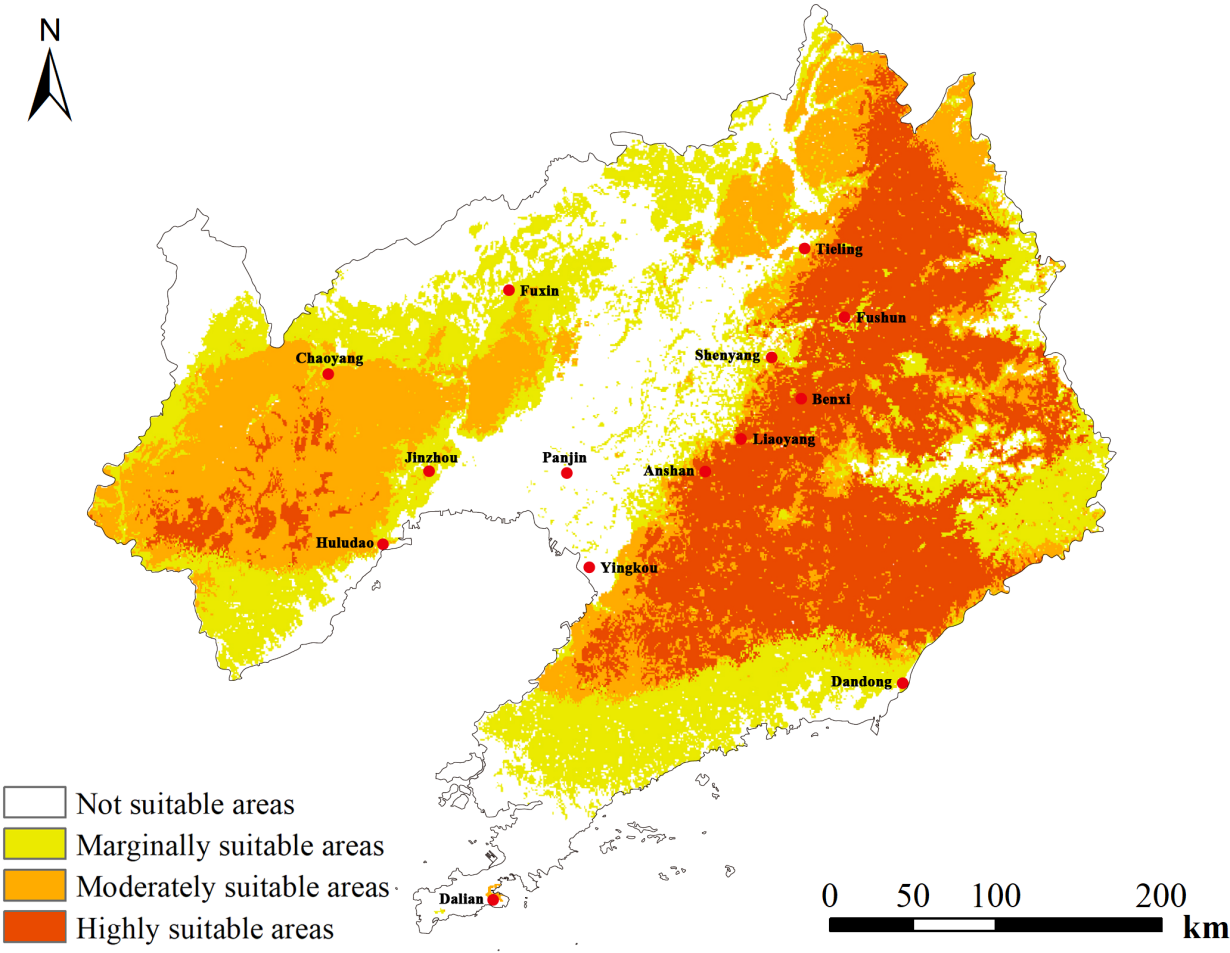
Predicted distribution of *D. dasycarpus* in Liaoning Province under current climate conditions.

Based on MaxEnt predictions for *D. dasycarpus* distribution, we next used ArcGIS software to perform reclassification and determine the most suitable potential areas for the distribution of *D. dasycarpus* in Liaoning Province (**Figure 4**). Analysis revealed that the total area of highly suitable and moderately suitable regions for *D. dasycarpus* was 7.082 × 10^4^ km^2^, accounting for 47.66% of the total area of Liaoning Province. The highly suitable area covered 3.468 × 10^4^ km^2^, and was primarily located in Tieling, Fushun, Liaoyang, Benxi, Anshan, Yingkou and the northern region of Dandong. The moderately suitable area spanned 3.614 × 10^4^ km^2^, encompassing Chaoyang, Huludao, Jinzhou, and other regions. The generally suitable area measured 3.873 × 10^4^ km^2^ and was distributed in Fuxin, the northern region of Dalian, and other areas. The unsuitable area amounted to 3.905 × 10^4^ km^2^ and was located in Panjin and Shenyang. Overall, several areas within Liaoning Province were identified to be suitable for the growth of *D. dasycarpus*, with the most suitable regions predominantly situated in the eastern part of the province. A smaller portion of suitable areas was identified in the western part of Liaoning Province. These findings indicated that the modern potential distribution areas predicted by the MaxEnt model were largely consistent with contemporary distribution records.

### 3.3 Geodetector analysis of the ecological suitability of *D. dasycarpus*

During the factor detection phase of Geodetector analysis, the q-value reflected the explanatory power of environmental variables on the ecological suitability of *D. dasycarpus*. A larger q-value indicated a stronger explanatory power. In this study, *p*<0.05 was used as the threshold to indicate that environmental variables had a significant effect on habitat suitability. Of these variables, Prec7 (July precipitation) exerted the largest impact on the spatial differentiation of ecological suitability in Liaoning Province, followed by Srad5 (May solar radiation) and Tmax3 (March maximum temperature). Bio4 (temperature seasonality) had the smallest impact (**Supplementary Table S3**).

During the interaction detection phase, the interactions between environmental variables exhibited an enhancement effect. The interaction between Prec7 and other environmental variables had the greatest impact on the spatial differentiation of habitat suitability (**Supplementary Table S4**). The combined effects of multiple variables were stronger than the individual effects of single variables. The interaction between Srad5 and Tmax3 also influenced the spatial differentiation of habitat suitability but to a lesser extent compared to the interaction between Prec7 and Bio4. All environmental variables intersected across different grading levels of ecologically suitable zones. Generally, the precipitation factor and comprehensive climate factors followed an upwards trend, while the temperature factor and radiation factor exhibited a downwards trend (**Table 2**). This pattern was consistent with the preference of *D. dasycarpus* for a warm and humid climate, its tolerance to cold, and its sensitivity to strong light.

**Table 2:** Information relating to the environmental variables corresponding to different ecological suitability levels.

Environment variables	Ecological suitability level		
	Marginal	Moderate	High
Bio4	99.53~130.94	118.91~131.67	113.18~132.93
Tmax3/°C	4.4~8.7	3.6~8.6	3.1~7.2
Srad5/kJ·m^-2^·d^-1^	20268~22124	20115~22098	20109~22068
Prec7/mm	158~228	161~258	179~317

### 3.4 Correlation analysis between environmental variables and chemical components

Results arising from the component analysis of *D. dasycarpus* are presented in **Supplementary Table S5**. Methodological validation revealed that the linear correlation coefficients (r) for limonin, dictamnine, obacunone, and fraxinellon were 0.9999, 0.9997, 0.9997 and 0.9999, respectively (**Supplementary Table S6**). The relative standard deviations (RSDs) for precision were 1.3%, 1.2%, 2.7%, and 1.2%, respectively; for stability, the RSDs were 2.5%, 2.9%, 2.9% and 2.8%; and for repeatability, the RSDs were 1.9%, 1.1%, 1.6% and 2.9%. The recovery rates were 98.53%, 97.26%, 98.83% and 96.37%, with corresponding RSDs of 2.8%, 1.3%, 2.3% and 2.1%. The results arising from grey correlation analysis are provided in **Supplementary Table S7**. Pearson correlation analysis (**Supplementary Table S8**) identified the environmental variables that exerted significant influence on pharmacodynamic components, and could be used to construct subsequent regression equations. Analysis revealed differences in the environmental variables affecting cultivated in comparison to wild samples.

### 3.5 Analysis of quality zoning for *D. dasycarpus*

Quality zoning analysis of limonin, dictamnine, obacunone, and fraxinellon in *D. dasycarpus* was conducted to investigate the spatial distribution and quality variations of these important bioactive compounds. Our findings indicated significant spatial heterogeneity in the concentration of these active components across different regions, with specific environmental factors playing a crucial role.

#### 3.5.1 Quality zoning analysis of limonin

**Figure 5:**
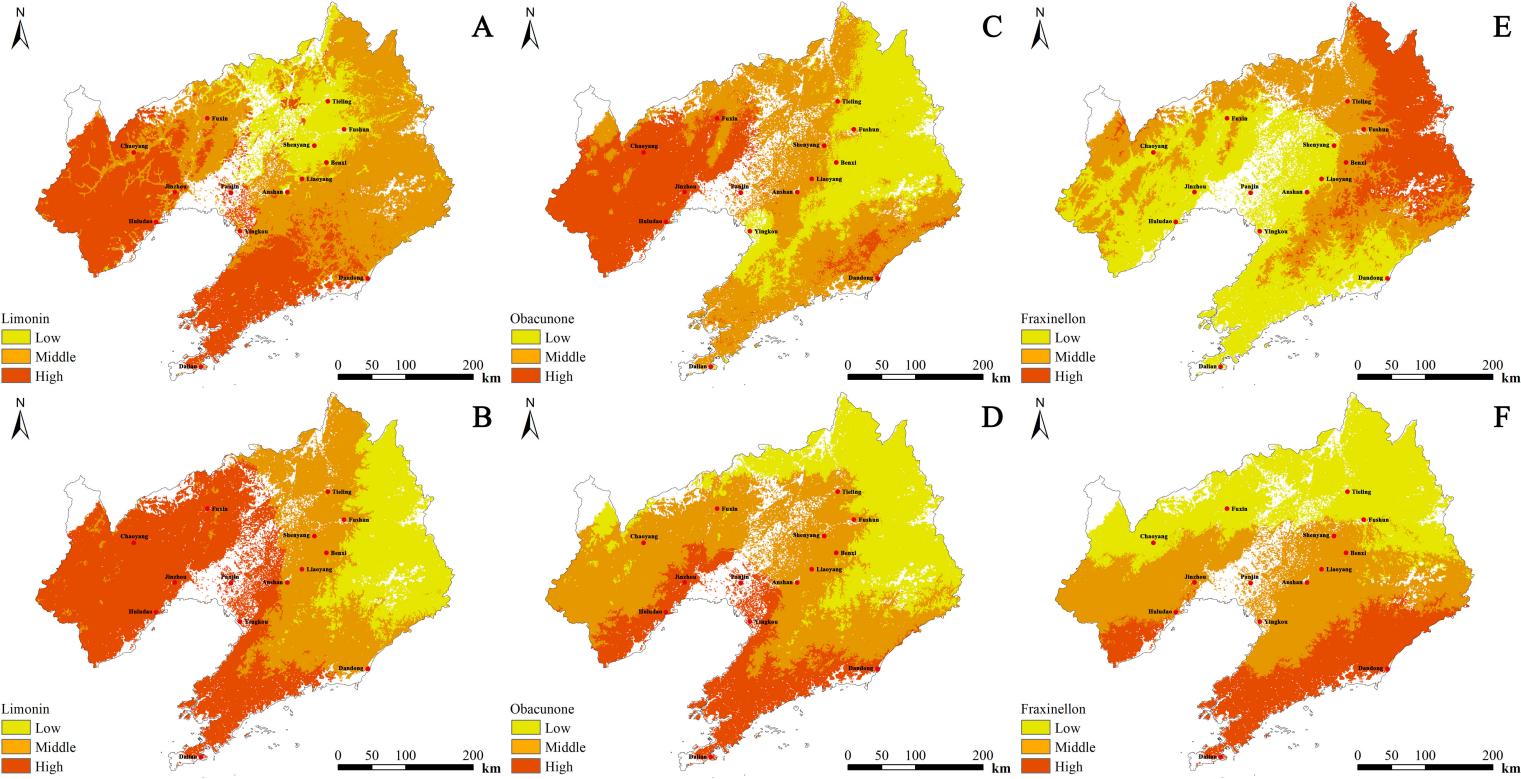
The spatial distribution of the content of *D*. *dasycarpus*. **(A)** Limonin content of cultivated samples. **(B)** Limonin content of wild samples. **(C)** Obacunone content of cultivated samples. **(D)** Obacunone content of wild samples. **(E)** Fraxinellon content of cultivated samples. **(F)** Fraxinellon content of wild samples.

The regression equation for limonin content (*Y*) in cultivated *D. dasycarpus* with respect to environmental variables was *Y*=0.717-0.009 Bio1-0.032 Tmean7 + 0.001 Srad3 + 0.082 Tmin3. Limonin content exhibited negative correlations with Tmean7 and Tmin3, and positive correlations with Bio1 and Srad3. This implies that limonin content in cultivated *D. dasycarpus* increases with higher annual mean temperature (Bio1) and greater solar radiation in March (Srad3), while it decreases as the mean temperature in July (Tmean7) or the minimum temperature in March (Tmin3) rises. High-quality zones were predominantly identified in the southwestern part of Liaoning Province, while the central region generally exhibited medium quality. Overall, limonin content decreased from the southwestern to the northeastern parts of Liaoning Province (**Figure 5A**).

The regression equation for limonin content (*Y*) in wild *D. dasycarpus* with respect to environmental variables was *Y*=3.692-0.029 Prec3 + 0.007 Tmean1. Limonin content exhibited a negative correlation with Prec3 and a positive correlation with Tmean1. This implies that limonin content in wild *D. dasycarpus* increases with higher mean temperature in January (Tmean1) and decreases with greater precipitation in March (Prec3). High-quality zones were mainly located in the western part of Liaoning Province, while medium-quality zones were distributed in the eastern regions. Overall, limonin content decreased from the western to the eastern parts of Liaoning Province (**Figure 5B**).

#### 3.5.2 Quality zoning analysis of obacunone

The regression equation for obacunone content (*Y*) in cultivated *D. dasycarpus* with respect to environmental variables was *Y*=-14.733 + 0.42 Bio1 + 0.105 index_wi-0.401 Tmax3 + 0.032 Bio15 + 0.083 Bio10 + 0.083 Bio16. Obacunone content exhibited a negative correlation with Bio16 and positive correlations with the other environmental variables. This implies that obacunone content in cultivated *D. dasycarpus* increases with higher annual mean temperature (Bio1), higher warmth index (index_wi), and more favorable values of Bio15 (precipitation seasonality), Bio10 (mean temperature of the warmest quarter), and Bio16 (precipitation of the warmest quarter), while it decreases as the maximum temperature in March (Tmax3) rises. High-quality zones were predominantly found in the western and southeastern parts of Liaoning Province, while the eastern and central regions generally exhibited medium quality. Overall, quality decreased from west to east in Liaoning Province (**Figure 5C**).

The regression equation for obacunone content (*Y*) in wild *D. dasycarpus* with respect to environmental variables was *Y*=4.544 + 0.024 Tmean12. Obacunone content exhibited a significant positive correlation with Tmean12. This implies that obacunone content in wild *D. dasycarpus* increases significantly with higher mean temperature in December (Tmean12). High-quality zones were mainly located in the southern and western parts of Liaoning Province, while medium-quality zones were distributed in the northeastern region. Overall, quality decreased from south to north in Liaoning Province (**Figure 5D**).

#### 3.5.3 Quality zoning analysis of fraxinellon

For cultivated *D. dasycarpus*, the regression equation for fraxinellon content (*Y*) with respect to environmental variables was *Y*=1.136-0.171 Tmean2-0.045 Prec4 + 0.086 Bio9 + 0.086 Bio11 + 0.068 Prec5. Fraxinellon content exhibited negative correlations with Bio9, Bio11 and Tmean2, but exhibited positive correlations with Prec4 and Prec5. This implies that fraxinellon content in cultivated *D. dasycarpus* increases with higher precipitation in April (Prec4) and May (Prec5) but decreases as the mean temperature in February (Tmean2), mean temperature of the driest quarter (Bio9), or mean temperature of the coldest quarter (Bio11) rises. High-quality zones were predominantly found in northeastern Liaoning Province, whereas central, southern, and western regions generally exhibited medium quality. Overall, quality decreased from the northeastern to the southwestern parts of Liaoning Province (**Figure 5E**).

For wild *D. dasycarpus*, the regression equation for fraxinellon content (*Y*) with respect to environmental variables was *Y*=3.974 + 0.04 Bio1 + 0.003 Prec7-0.03 Tmean6. Fraxinellon content exhibited negative correlations with Tmean6, but exhibited positive correlations with Prec7 and Bio1. This implies that fraxinellon content in wild *D. dasycarpus* increases with higher annual mean temperature (Bio1) and greater July precipitation (Prec7) but decreases as the mean temperature in June (Tmean6) rises. High-quality zones were mainly located in the southern and western parts of Liaoning Province, while medium-quality zones were distributed in the northern regions. Overall, quality decreased from the southern to the northern parts of Liaoning Province (**Figure 5F**).

#### 3.5.4 Quality zoning analysis of dictamnine

Following comprehensive analysis and calculation, no significant correlation was identified between the dictamnine content in both cultivated and wild *D. dasycarpus* and environmental variables. Consequently, it was not possible to construct a regression equation to model this relationship, indicating that environmental variables did not exert significant influence on the content of dictamnine.

### 3.6 Comprehensive quality zoning of *D. dasycarpus*

We detected variable correlations between the same environmental variable and different pharmacological components, as well as between different environmental variables and the same pharmacological component. We addressed this issue by overlaying the results of three single-index quality zoning analyses in ArcGIS software. This process yielded a comprehensive set of quality zoning results for *D. dasycarpus* in Liaoning Province.

**Figure 6:**
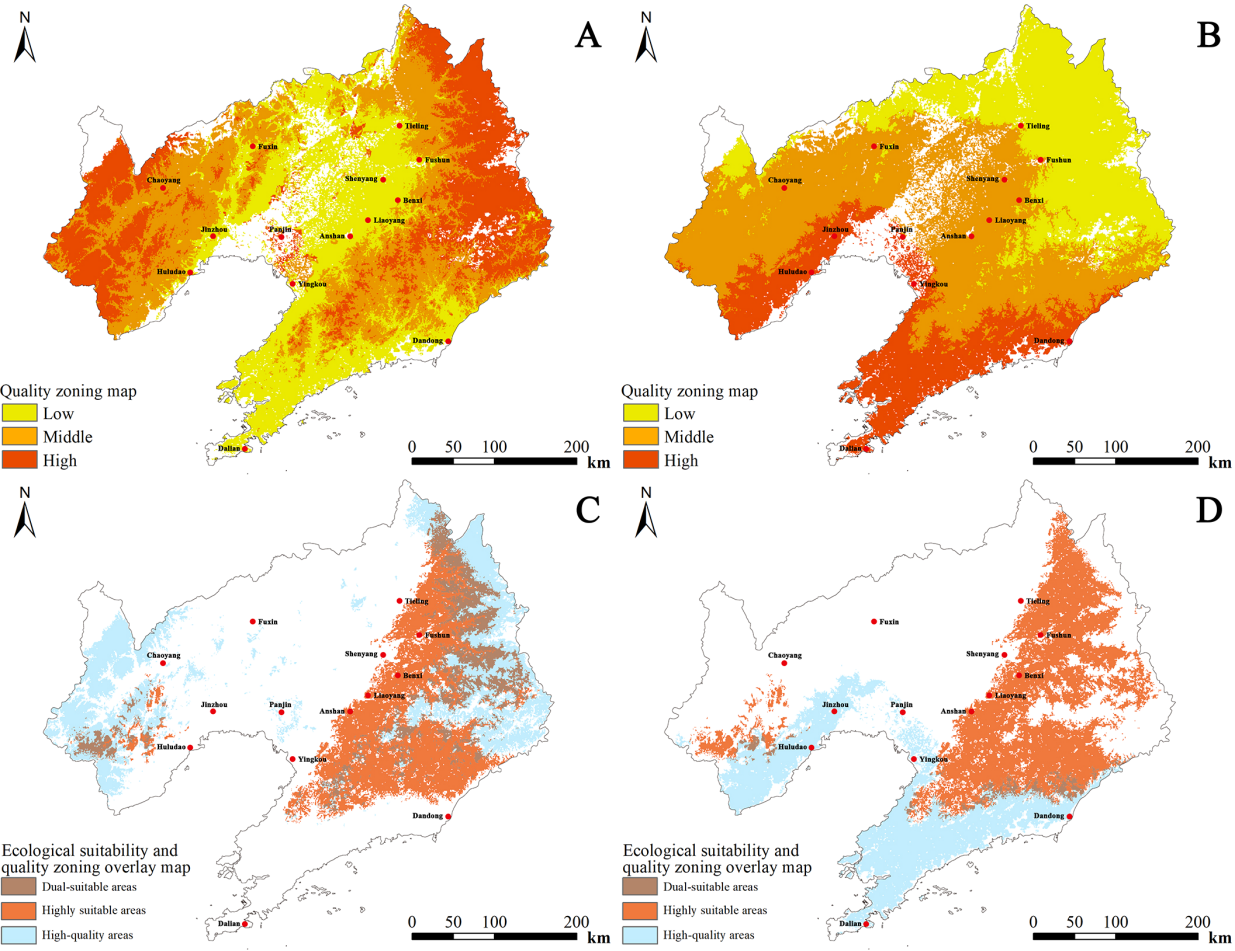
Comprehensive quality zoning and ecological suitability overlay maps of *D. dasycarpus*. **(A)** Cultivated *D*. *dasycarpus*. **(B)** Wild *D*. *dasycarpus*. **(C)** Overlay of high-quality and highly suitable areas for cultivated *D*. *dasycarpus*. **(D)** Overlay of high-quality and highly suitable areas for wild *D*. *dasycarpus*.

For cultivated *D. dasycarpus*, the high-quality zone covered an area of 3.710 × 10^4^ km^2^, accounting for 24.97% of the total area of Liaoning Province. The high-quality zone was primarily concentrated in eastern regions, including Tieling, Fushun, and Benxi, as well as western regions, such as Chaoyang and Huludao. The medium-quality zone spanned 4.932 × 10^4^ km^2^ and was distributed in parts of Dandong, Tieling, Benxi in the east, and Fuxin in the west. The general-quality zone occupied 4.405 × 10^4^ km^2^ and was mainly located in central areas such asShenyang, Liaoyang, and Anshan, as well as southern areas, including Yingkou and Dalian. Overall, quality decreased from the western and eastern parts towards the central part of Liaoning (**Figure 6A**).

For wild *D. dasycarpus*, the high-quality zone covered 2.647 × 10^4^ km^2^, representing 17.82% of the total area of the province. This area was predominantly found in southern regions such as Dalian and western regions, including Huludao. The medium-quality zone spanned 6.167 × 10^4^ km^2^ and was concentrated in areas such as Chaoyang, Jinzhou, Shenyang, Anshan, Liaoyang, and Dandong. The general-quality zone occupied 4.232 × 10^4^ km^2^ and was mainly located in northern regions such as Tieling and Fushun. Overall, quality decreased from the south to the north of Liaoning (**Figure 6B**).

### 3.7 Geodetector analysis for the comprehensive quality of *D. dasycarpus*

Factor detection in Geodetector analysis demonstrated that Tmean2 and Prec5 were the environmental variables that had the greatest impact on the spatial heterogeneity of the quality of cultivated *D. dasycarpus* (**Supplementary Table S9**). For wild *D. dasycarpus*, Tmean1 had the most significant impact on spatial heterogeneity, followed by Tmean12 and Bio1 (**Supplementary Table S10**). In terms of interaction detection, the interactions between environmental variables affecting the quality of cultivated *D. dasycarpus* all exhibited enhancing effects, with the interaction between Prec5 and Tmean2 being particularly significant (**Supplementary Table S11**). For wild *D. dasycarpus*, all interactions were two-factor enhancements, with the interaction between Tmean1 and Bio1 being significant. Other non-primary environmental variables, such as the interaction between precipitation factors (Prec7) and temperature factors (Tmean1, Tmean12), also played a role (**Supplementary Table S12**). Each environmental variable overlapped across different quality grades. For cultivated *D. dasycarpus*, precipitation factors followed an increasing trend, while temperature factors followed a decreasing trend; for wild *D. dasycarpus*, temperature factors also followed a decreasing trend (**Table 3**).

**Table 3:** Information relating to environmental variables corresponding to different quality levels of cultivated *D. dasycarpus* and wild *D. dasycarpus*.

Ecotype	Environment variables	Comprehensive quality level		
		Low	Mid	High
Cultivation	Prec5/mm	35~58	40~67	62~73
	Tmean2/°C	-8.4~-6.5	-9.6~-6.0	-11.7~-10.0
Wild	Bio1/°C	7.7~10.6	6.2~8.0	4.8~7.0
	Tmean1/°C	-10.6~-4.8	-11.9~-10.6	-15.8~-11.8

## 4 Discussion

*D. dasycarpus* is one of the principal medicinal herbs cultivated in Liaoning Province. Because its medicinal value has increasingly been recognized, wild resources of *D. dasycarpus* have been extensively exploited. Concurrently, rapid industrial development has led to habitat destruction, resulting in a year-on-year decline in wild resources and production, coupled with rising prices (Zhi et al., 2022). Under these circumstances, enhancing the quality of cultivated *D. dasycarpus* is of paramount importance. Therefore, conducting research on the ecological suitability and quality distribution of *D. dasycarpus* in Liaoning Province holds significant practical implications for identifying potential areas suitable for artificial cultivation and promoting the development of the medicinal herb industry.

*D. dasycarpus* exhibits biological characteristics that favor warm and humid climates, being cold-tolerant but intolerant to drought, waterlogging, and strong light. This herb typically grows on slopes with a certain gradient, in shrublands, grasslands, or under sparse forests, preferring higher altitudes with sufficient sunlight, well-drained soil rich in humus, and sandy loam or loam soils (Wang et al., 2023). Previous studies demonstrated that the optimal growth temperature for *D. dasycarpus* was between 12°C and 18°C (Wang, 2021). Extreme temperatures could reduce plant photosynthetic activity, affect protein stability, lead to the excessive accumulation of reactive oxygen species (ROS), and disrupt the production of plant hormones and signal transmission, thereby exerting adverse effects on the growth, development and quality of plants (Li et al., 2018; Ohama et al., 2017; Huang et al., 2023). In addition, water availability can exert significant effects on the emergence and growth of seedlings (Khaeim et al., 2022). Long-term drought could limit the growth and development, of plants, reduce photosynthetic intensity, and reduce the accumulation of secondary metabolites (Yang et al., 2007; Chaves et al., 2009; He et al., 2021). The climate of Liaoning Province is characterized by a temperate monsoon climate, marked by warm and rainy summers, cold and dry winters, distinct seasons, and significant temperature differences between the north and south. Field investigations have revealed that the central part of Liaoning Province has lower terrain and predominantly black soil and saline-alkali soil, making it less suitable for cultivating *D. dasycarpus*. In contrast, the western and eastern parts of Liaoning Province, with higher altitudes, ample sunlight and sandy loam soil, are more conducive to its growth.

We consulted information on iplant (www.iPlant.cn) which indicated that *D. dasycarpus* is distributed in certain cities, including Shenyang, Dalian, Anshan, Fushun, Jinzhou, Tieling and Chaoyang. Combining this information with results arising from the fourth national survey of TCM resources, our findings align with the predicted distribution of areas with ecological suitability for *D. dasycarpus*, thus validating the accuracy and rationality of our study.

The seeds of *D. dasycarpus* exhibit physiological after-ripening characteristics (Fu and Yang, 2021), and therefore requires vernalization for germination. The optimal sowing time is in April, coinciding with the transition of the Northern Hemisphere from winter to spring when temperatures gradually rise. In the most suitable areas, Tmax3 ranges from 3.1°C to 7.2°C, meeting the temperature requirements for seed germination and seedling growth. The flowering period occurs around May, during which light sensitivity is highest, making Srad5 a significant environmental variable. The fruiting period, which extends from July to August, requires substantial water. During this critical phase, Prec7 plays a pivotal role in ensuring sufficient moisture and nutrients for the development of vegetative organs such as the stems and leaves, as well as for the formation of fully mature fruits.

After comparing and analyzing the distribution results for ecological suitability and comprehensive quality, it is evident that low-quality areas do not align with ecological suitability zones. This indicates that environmental factors that are unfavorable to the growth of *D. dasycarpus* also inhibit the accumulation of medicinal components. However, this does not imply that all suitable areas are necessarily high-quality areas. The current distribution of cultivated *D. dasycarpus* quality largely corresponds to the areas that were predicted to be suitable. Variations in water, temperature and soil fertilizer management practices across different regions contribute to inconsistencies in the quality of medicinal material. In contrast, there are notable differences between the high-quality areas of wild *D. dasycarpus* and the suitable areas.

Studies have shown that the optimal conditions for the growth and development of medicinal plants and the accumulation of secondary metabolites may differ (Cheng et al., 2024). This discrepancy can be attributed to the complex nature of secondary metabolite synthesis and accumulation in medicinal plants. Since active compounds in medicinal plants are primarily secondary metabolites, these are often considered adaptations to adverse environmental stressors (Al-Khayri et al., 2023). Therefore, we hypothesize that the formation and accumulation of effective components occur under different environmental conditions than those required for optimal growth.

Field investigations have revealed that cultivated *D. dasycarpus* exhibits regular morphology, enlarged main roots, numerous lateral and fibrous roots, light color, smooth skin, firm texture, a strong powdered structure in cross-section, and a mild, sweet taste. In contrast, wild *D. dasycarpus* exhibits uneven main roots, twisted morphology, dark color, rough skin, spongy texture, strong fibrousness in cross-section, and a strong smell. Studies have indicated that wild medicinal plants experience more external stressors during growth. Salt stress and drought stress have been shown to slow plant growth, thicken the periderm to reduce water loss, and increase the xylem vessels to facilitate water distribution between above-ground and under-ground parts (Wang et al., 2024b). Furthermore, salt and drought stress can cause collapse of the cell wall and shrinkage of the roots, thus resulting in the generation of cavities in the phloem of plant roots, damage to root hairs, and a reduced trend for the main roots to absorb water. These factors ultimately contribute to specific characteristics in wild medicinal plants, including thin and long roots, a spongy texture, and strong fibrousness. Stress and the growth of duration can jointly exert influence on the content or proportion of secondary metabolites in wild medicinal plants, thus leading to their distinctive or intense odors.

In contrast, cultivated medicinal plants grow in artificially created suitable environments with weaker stress intensity than wild medicinal plants. Factors such as an abundance of water and fertilizer application, loose soil, and reduced ecological competition can promote vigorous primary metabolism, leading to the accumulation of large amounts of primary metabolites, including sucrose and starch. This results in certain characteristics, including enlarged main roots, uniform size, firm texture, flat cross-sections, weak fibrousness and an increased powdered texture (Wang et al., 2024a).

In the present study, geographical detector analysis revealed that precipitation and temperature factors exerted joint influence on the ecological suitability and quality of *D. dasycarpus*. Precipitation is the dominant environmental variable for ecological suitability analysis, while temperature is the dominant variable for quality analysis. Based on our findings, we hypothesize that water affects the growth of *D. dasycarpus*, whereas temperature influences the accumulation of its effective components.

Finally, by integrating the ecological suitability distribution area with the quality distribution area of wild *D. dasycarpus* and comparing this with current planting areas, we identified potential cultivation areas that were beyond the original planting zones. These included the southern part of Chaoyang, northern and western Huludao, western Jinzhou, eastern Liaoyang, and the contiguous eastern regions of Tieling, Fushun, and Benxi (**Figure 6C**), as well as western Dandong (**Figure 6D**). The ecological environment and quality of these areas are highly suitable, rendering these regions as highly likely candidates for potential cultivation. Nevertheless, in real cultivation scenarios, factors including pest infestations and inconsistencies in management strategies can give rise to discrepancies between predicted and observed results. Future research should prioritize expanding sampling coverage across diverse geographical and ecological gradients to enhance model generalizability. Additionally, integrating additional abiotic and biotic factors could substantially improve model precision (Comia-Geneta et al., 2024). We recommend validating model predictions through rigorous long-term monitoring and controlled field experiments, particularly in identified priority cultivation zones. Furthermore, incorporating plant breeding advancements into species distribution models (Li et al., 2024) would enable more robust projections under climate change scenarios. Such multidisciplinary approaches will facilitate the development of adaptive spatial management strategies that simultaneously address ecological suitability, phytochemical optimization, and agricultural sustainability.

The MaxEnt-HPLC-Geodetector integrated framework constructed in this study has the potential for cross-regional and cross-species application. The MaxEnt model, adept at handling the nonlinear species-environment relationship and adaptable to small sample data, can be transferred to predict ecological suitability in different regions; HPLC, as a standardized detection technology, has a component quantification method that can be directly applied to the quality assessment of other medicinal species; the Geodetector, by quantifying the contribution of environmental factors and their interaction effects, can analyze the spatial driving mechanism of quality formation. This framework, through the three-dimensional synergy of ecological distribution prediction (MaxEnt)–quality index correlation (HPLC)–driving factor analysis (Geodetector), provides a universal paradigm for the study of the authenticity of traditional Chinese medicinal materials and the protection of biological resources. In practical applications, the environmental variable set and chemical indicators need to be adjusted according to the target species, and model parameters need to be optimized to achieve regional adaptation and precise application of the methodology.

## 5 Conclusion

In this study, we utilized ArcGIS, the MaxEnt model, and HPLC techniques to analyze how environmental variables impact the ecologically suitable distribution and quality of *D. dasycarpus*. Analysis revealed that Prec7, Bio4, Srad5, Tmax3, and Tmin3 were key variables for the ecological distribution of *D. dasycarpus* and could influence growth areas in Liaoning. Tmean2 and Prec5 were identified as being important for the quality of cultivated *D. dasycarpus*, while Tmean1 was identified to be crucial for quality of the wild variety. Model analysis determined suitable distribution areas in Liaoning; eastern cities such as Tieling, Fushun, and Liaoyang were considered to be ideal. High-quality cultivated *D. dasycarpus* were found to be concentrated in Tieling (east) and Chaoyang (west), while wild plants were focussed in Dalian (south) and Huludao (west). Geodetector analysis demonstrated that environmental variables have dominant, phased and comprehensive effects on ecological suitability and quality, with variable interactions also significant. Integrating ecological and quality results provided theoretical support for selecting high-quality planting areas, thus facilitating the protection on wild resources, optimizing planting layout, increasing both yield and quality, and promoting industry sustainability. Our study innovatively integrates the MaxEnt model, HPLC, and Geodetector to establish a comprehensive research framework capable of predicting species’ ecological suitability and quality distribution, providing a reference for related studies on other species and regions. This approach is expected to promote the transformation of this field from single-component research to comprehensive ecological pharmacology research, providing new ideas and methods for ecology-based quality control of medicinal materials and the sustainable utilization of resources.

## Data Availability

The original contributions presented in the study are included in the article/**Supplementary Material**. Further inquiries can be directed to the corresponding author.
