# Supplementary material for: Construction of eco-quality dual-suitable distribution areas for Dictamnus dasycarpus Turcz.: integrated analysis of MaxEnt model and multidimensional indicators
Source: Front Plant Sci. 2025 Jul 16;16:1591921. doi: 10.3389/fpls.2025.1591921 (PMC12307492; doi:10.3389/fpls.2025.1591921)
Supplement: Supplementary file 1 [file Table1.docx]

***Supplementary Material***

**Table S1.** Sampling point information *D. dasycarpus*.

| NO. | longtitude | latitude | Ecotype | NO. | longtitude | latitude | Ecotype |
| --- | --- | --- | --- | --- | --- | --- | --- |
| ZP-1 | 123.141111 | 41.2525 | Cultivation | YS-1 | 119.920723 | 41.519463 | Wild |
| ZP-2 | 125.087625 | 42.177649 | Cultivation | YS-2 | 118.931903 | 40.80096 | Wild |
| ZP-3 | 124.997206 | 42.133757 | Cultivation | YS-3 | 119.731392 | 40.782237 | Wild |
| ZP-4 | 125.098922 | 42.155276 | Cultivation | YS-4 | 120.237222 | 40.84219 | Wild |
| ZP-5 | 124.872845 | 42.350528 | Cultivation | YS-5 | 124.322494 | 40.315428 | Wild |
| ZP-6 | 124.856245 | 42.384635 | Cultivation | YS-6 | 121.678557 | 41.583125 | Wild |
| ZP-7 | 124.851657 | 41.712824 | Cultivation | YS-7 | 125.539665 | 41.309555 | Wild |
| ZP-8 | 125.016722 | 42.146986 | Cultivation | YS-8 | 123.454684 | 41.25416 | Wild |
| ZP-9 | 124.80444 | 42.416365 | Cultivation | YS-9 | 123.292093 | 41.121157 | Wild |
| ZP-10 | 124.630312 | 42.58082 | Cultivation | YS-10 | 123.252396 | 40.889755 | Wild |
| ZP-11 | 123.892678 | 42.140997 | Cultivation | YS-11 | 123.228388 | 41.1394 | Wild |
| ZP-12 | 125.367544 | 41.272925 | Cultivation | YS-12 | 123.142063 | 41.051174 | Wild |
| ZP-13 | 125.425893 | 41.596233 | Cultivation | YS-13 | 123.084453 | 40.972576 | Wild |
| ZP-14 | 124.077425 | 41.392332 | Cultivation | YS-14 | 123.579369 | 40.326112 | Wild |
| ZP-15 | 125.035392 | 41.392652 | Cultivation | YS-15 | 123.135 | 41.006318 | Wild |
| ZP-16 | 124.241057 | 41.46014 | Cultivation | YS-16 | 124.79029 | 40.70975 | Wild |
| ZP-17 | 125.036442 | 41.391475 | Cultivation | YS-17 | 121.504042 | 38.87244 | Wild |
| ZP-18 | 124.067242 | 41.375158 | Cultivation | YS-18 | 121.94304 | 39.075878 | Wild |
| ZP-19 | 119.781851 | 41.167306 | Cultivation | YS-19 | 123.646081 | 41.928575 | Wild |
| ZP-20 | 120.097112 | 41.368315 | Cultivation | YS-20 | 125.245108 | 41.646812 | Wild |
| ZP-21 | 123.001944 | 40.7075 | Cultivation | YS-21 | 124.640512 | 41.400383 | Wild |
| ZP-22 | 122.932274 | 40.796445 | Cultivation | YS-22 | 124.816291 | 42.031469 | Wild |
| ZP-23 | 120.130465 | 40.762375 | Cultivation | YS-23 | 123.69321 | 41.140769 | Wild |
| ZP-24 | 124.531978 | 40.52192 | Cultivation | YS-24 | 124.737505 | 42.783401 | Wild |
| ZP-25 | 124.79029 | 40.700975 | Cultivation |  |  |  |  |

**Table S2.** 106 environmental variables.

| NO. | Abbreviated name | Variable description | Unit |
| --- | --- | --- | --- |
| 1 | altitude | altitude | m |
| 2 | Aspect | Aspect | ° |
| 3 | Slope | Slope | ° |
| 4 | zblx | Vegetation type | — |
| 5 | index_ci | Cold index | — |
| 6 | index_hi | Humidity index | — |
| 7 | index_wi | Warmth index | — |
| 8 | pH | Soil pH | — |
| 9 | YJTHL | Organic carbon content in the upper layer of soil (0-30cm) | % |
| 10 | HSL | Sediment content in the upper layer of the soil (0-30cm) | % |
| 11 | NTL | Clay content in the upper layer of the soil (0-30cm) | % |
| 12 | TRYXHSLDJ | Grade of soil available water content | — |
| 13 | SoilType | SoilType | — |
| 14 | TRZDFL | Soil texture classification | — |
| 15 | TRYLZJHNL | Cation exchange capacity in the upper layer of soil (0-30cm) | c mol/kg |
| 16 | prec1 | Average precipitation in January | mm |
| 17 | prec2 | Average precipitation in February | mm |
| 18 | prec3 | Average precipitation in March. | mm |
| 19 | prec4 | Average precipitation in April | mm |
| 20 | prec5 | Average precipitation in May. | mm |
| 21 | prec6 | Average precipitation in June | mm |
| 22 | prec7 | Average precipitation in July | mm |
| 23 | prec8 | Average precipitation in August | mm |
| 24 | prec9 | Average precipitation in September | mm |
| 25 | prec10 | Average precipitation in October | mm |
| 26 | prec11 | Average precipitation in November. | mm |
| 27 | prec12 | Average precipitation in November. | mm |
| 28 | tmean1 | Average temperature in January | ℃ |
| 29 | tmean2 | Average temperature in February | ℃ |
| 30 | tmean3 | Average temperature in March. | ℃ |
| 31 | tmean4 | Average temperature in April | ℃ |
| 32 | tmean5 | Average temperature in May | ℃ |
| 33 | tmean6 | Average temperature in June | ℃ |
| 34 | tmean7 | Average temperature in July | ℃ |
| 35 | tmean8 | Average temperature in August | ℃ |
| 36 | tmean9 | Average temperature in September | ℃ |
| 37 | tmean10 | Average temperature in October | ℃ |
| 38 | tmean11 | Average temperature in November | ℃ |
| 39 | tmean12 | Average temperature in December | ℃ |
| 40 | Tmax1 | Maximum temperature in January | ℃ |
| 41 | Tmax2 | Maximum temperature in February | ℃ |
| 42 | Tmax3 | Maximum temperature in March. | ℃ |
| 43 | Tmax4 | Maximum temperature in April | ℃ |
| 44 | Tmax5 | Maximum temperature in May | ℃ |
| 45 | Tmax6 | Maximum temperature in June | ℃ |
| 46 | Tmax7 | Maximum temperature in July | ℃ |
| 47 | Tmax8 | Maximum temperature in August | ℃ |
| 48 | Tmax9 | Maximum temperature in September | ℃ |
| 49 | Tmax10 | Maximum temperature in October | ℃ |
| 50 | Tmax11 | Maximum temperature in November | ℃ |
| 51 | Tmax12 | Maximum temperature in December | ℃ |
| 52 | Tmin1 | Minimum temperature in January | ℃ |
| 53 | Tmin2 | Minimum temperature in February | ℃ |
| 54 | Tmin3 | Minimum temperature in March. | ℃ |
| 55 | Tmin4 | Minimum temperature in April | ℃ |
| 56 | Tmin5 | Minimum temperature in May | ℃ |
| 57 | Tmin6 | Minimum temperature in June | ℃ |
| 58 | Tmin7 | Minimum temperature in July | ℃ |
| 59 | Tmin8 | Minimum temperature in August | ℃ |
| 60 | Tmin9 | Minimum temperature in September | ℃ |
| 61 | Tmin10 | Minimum temperature in October | ℃ |
| 62 | Tmin11 | Minimum temperature in November | ℃ |
| 63 | Tmin12 | Minimum temperature in December | ℃ |
| 64 | Srad1 | Solar radiation in January | kJ m^-2^ day^-1^ |
| 65 | Srad2 | Solar radiation in February | kJ m^-2^ day^-1^ |
| 66 | Srad3 | Solar radiation in March. | kJ m^-2^ day^-1^ |
| 67 | Srad4 | Solar radiation in April | kJ m^-2^ day^-1^ |
| 68 | Srad5 | Solar radiation in May | kJ m^-2^ day^-1^ |
| 69 | Srad6 | Solar radiation in June | kJ m^-2^ day^-1^ |
| 70 | Srad7 | Solar radiation in July | kJ m^-2^ day^-1^ |
| 71 | Srad8 | Solar radiation in August | kJ m^-2^ day^-1^ |
| 72 | Srad9 | Solar radiation in September | kJ m^-2^ day^-1^ |
| 73 | Srad10 | Solar radiation in October | kJ m^-2^ day^-1^ |
| 74 | Srad11 | Solar radiation in November | kJ m^-2^ day^-1^ |
| 75 | Srad12 | Solar radiation in December | kJ m^-2^ day^-1^ |
| 76 | Vapr1 | Water vapor pressure in January | kPa |
| 77 | Vapr2 | Water vapor pressure in February | kPa |
| 78 | Vapr3 | Water vapor pressure in March. | kPa |
| 79 | Vapr4 | Water vapor pressure in April | kPa |
| 80 | Vapr5 | Water vapor pressure in May | kPa |
| 81 | Vapr6 | Water vapor pressure in June | kPa |
| 82 | Vapr7 | Water vapor pressure in July | kPa |
| 83 | Vapr8 | Water vapor pressure in August | kPa |
| 84 | Vapr9 | Water vapor pressure in September | kPa |
| 85 | Vapr10 | Water vapor pressure in October | kPa |
| 86 | Vapr11 | Water vapor pressure in November | kPa |
| 87 | Vapr12 | Water vapor pressure in December | kPa |
| 88 | BIO1 | Annual Mean Temperature | ℃ |
| 89 | BIO2 | Mean Diurnal Range (Mean of monthly (max temp - min temp)) | ℃ |
| 90 | BIO3 | Isothermality (BIO2/BIO7) (* 100) | — |
| 91 | BIO4 | Temperature Seasonality (standard deviation *100) | — |
| 92 | BIO5 | Max Temperature of Warmest Month | ℃ |
| 93 | BIO6 | Min Temperature of Coldest Month | ℃ |
| 94 | BIO7 | Temperature Annual Range (BIO5-BIO6) | ℃ |
| 95 | BIO8 | Mean Temperature of Wettest Quarter | ℃ |
| 96 | BIO9 | Mean Temperature of Driest Quarter | ℃ |
| 97 | BIO10 | Mean Temperature of Warmest Quarter | ℃ |
| 98 | BIO11 | Mean Temperature of Coldest Quarter | ℃ |
| 99 | BIO12 | Annual Precipitation | mm |
| 100 | BIO13 | Precipitation of Wettest Month | mm |
| 101 | BIO14 | Precipitation of Driest Month | mm |
| 102 | BIO15 | Precipitation Seasonality (Coefficient of Variation) | — |
| 103 | BIO16 | Precipitation of Wettest Quarter | mm |
| 104 | BIO17 | Precipitation of Driest Quarter | mm |
| 105 | BIO18 | Precipitation of Warmest Quarter | mm |
| 106 | BIO19 | Precipitation of Coldest Quarter | mm |

**Table S3.** Results of factor detection of main environmental variables.

| Environment variables | q | *p* |
| --- | --- | --- |
| Bio4 | 0.145 | 0.000 |
| Prec7 | 0.310 | 0.000 |
| Tmax3 | 0.279 | 0.000 |
| Srad5 | 0.308 | 0.000 |

**Table S4**. Results of interaction detection of main ecological factors.

|  | Bio4 | Prec7 | Tmax3 | Srad5 |
| --- | --- | --- | --- | --- |
| Bio4 | 0.145 |  |  |  |
| Prec7 | 0.533 | 0.310 |  |  |
| Tmax3 | 0.425 | 0.465 | 0.279 |  |
| Srad5 | 0.517 | 0.504 | 0.449 | 0.318 |

**Table S5. Determination and calculation results for 4 indicators in 49 sampling points.**

| NO. | Limonin (mg/g) | Dictamnine (mg/g) | Obacunone (mg/g) | Fraxinellon (mg/g) | Ecotype |
| --- | --- | --- | --- | --- | --- |
| ZP-1 | 2.175 | 0.309 | 2.809 | 0.852 | Cultivation |
| ZP-2 | 2.429 | 0.277 | 3.980 | 0.625 | Cultivation |
| ZP-3 | 5.023 | 0.385 | 4.997 | 1.021 | Cultivation |
| ZP-4 | 3.783 | 0.471 | 3.311 | 1.666 | Cultivation |
| ZP-5 | 2.737 | 0.426 | 4.424 | 0.637 | Cultivation |
| ZP-6 | 1.761 | 0.156 | 3.037 | 1.037 | Cultivation |
| ZP-7 | 1.864 | 0.600 | 1.812 | 1.768 | Cultivation |
| ZP-8 | 1.651 | 0.535 | 2.534 | 0.663 | Cultivation |
| ZP-9 | 3.181 | 0.534 | 1.011 | 1.908 | Cultivation |
| ZP-10 | 3.539 | 0.522 | 1.179 | 1.987 | Cultivation |
| ZP-11 | 2.465 | 0.148 | 5.600 | 0.432 | Cultivation |
| ZP-12 | 3.376 | 0.460 | 3.306 | 1.511 | Cultivation |
| ZP-13 | 2.591 | 0.349 | 3.119 | 0.567 | Cultivation |
| ZP-14 | 3.516 | 0.542 | 4.737 | 0.616 | Cultivation |
| ZP-15 | 2.324 | 0.382 | 0.858 | 1.077 | Cultivation |
| ZP-16 | 2.688 | 0.235 | 3.410 | 0.804 | Cultivation |
| ZP-17 | 4.905 | 0.801 | 1.474 | 1.548 | Cultivation |
| ZP-18 | 2.548 | 0.774 | 1.310 | 1.933 | Cultivation |
| ZP-19 | 2.491 | 0.370 | 1.153 | 1.187 | Cultivation |
| ZP-20 | 3.293 | 0.475 | 1.952 | 1.119 | Cultivation |
| ZP-21 | 4.058 | 0.468 | 2.586 | 1.757 | Cultivation |
| ZP-22 | 3.279 | 0.367 | 2.695 | 0.839 | Cultivation |
| ZP-23 | 3.085 | 0.402 | 2.690 | 0.683 | Cultivation |
| ZP-24 | 3.019 | 0.517 | 1.417 | 1.488 | Cultivation |
| ZP-25 | 1.339 | 0.462 | 1.636 | 2.708 | Cultivation |
| YS-1 | 2.119 | 0.369 | 1.945 | 1.144 | Wild |
| YS-2 | 2.334 | 0.328 | 2.984 | 0.795 | Wild |
| YS-3 | 4.028 | 0.656 | 3.292 | 1.771 | Wild |
| YS-4 | 2.327 | 0.303 | 2.349 | 1.162 | Wild |
| YS-5 | 4.033 | 0.759 | 3.220 | 1.631 | Wild |
| YS-6 | 3.789 | 0.497 | 2.803 | 1.168 | Wild |
| YS-7 | 2.428 | 0.233 | 1.437 | 2.961 | Wild |
| YS-8 | 2.557 | 0.569 | 1.158 | 1.209 | Wild |
| YS-9 | 2.853 | 0.254 | 2.276 | 2.418 | Wild |
| YS-10 | 2.991 | 0.417 | 3.324 | 1.060 | Wild |
| YS-11 | 1.935 | 0.329 | 2.340 | 1.831 | Wild |
| YS-12 | 2.127 | 0.284 | 2.453 | 0.661 | Wild |
| YS-13 | 2.602 | 0.338 | 1.493 | 1.241 | Wild |
| YS-14 | 2.895 | 0.490 | 1.393 | 1.377 | Wild |
| YS-15 | 1.508 | 0.272 | 2.152 | 1.012 | Wild |
| YS-16 | 1.094 | 0.332 | 2.028 | 1.699 | Wild |
| YS-17 | 2.831 | 0.458 | 4.017 | 2.189 | Wild |
| YS-18 | 1.492 | 0.563 | 6.211 | 3.551 | Wild |
| YS-19 | 3.714 | 0.460 | 3.368 | 1.371 | Wild |
| YS-20 | 1.364 | 0.102 | 3.452 | 0.802 | Wild |
| YS-21 | 1.487 | 0.250 | 2.772 | 0.867 | Wild |
| YS-22 | 1.200 | 0.220 | 1.803 | 0.489 | Wild |
| YS-23 | 2.164 | 0.335 | 3.160 | 2.621 | Wild |
| YS-24 | 1.926 | 0.322 | 2.153 | 0.588 | Wild |

**Table S6.** Results of linear relationship investigation.

| Chemical composition | Regression equation | r | linearity range（μg） |
| --- | --- | --- | --- |
| Limonin | *Y* = 4251.8 *X* - 1.4841 | 0.9999 | 2.50~60.00 |
| Dictamnine | *Y* = 38603 *X* - 31.633 | 0.9997 | 2.50~60.00 |
| Obacunone | *Y* = 15706 *X* + 2.9929 | 0.9997 | 2.50~60.00 |
| Fraxinellon | *Y* = 14906 *X* - 3.0847 | 0.9999 | 1.25~40.00 |

**Table S7.** Results of grey correlation analysis.

| Chemical composition | Environment variables | Degree of correlation | Ranking | Ecotype |
| --- | --- | --- | --- | --- |
| Limonin | hsl | 0.919 | 1 | Cultivation |
|  | ntl | 0.918 | 2 |  |
|  | tmean7 | 0.912 | 3 |  |
|  | tryxshldj | 0.912 | 4 |  |
|  | bio1 | 0.912 | 5 |  |
|  | tmin03 | 0.912 | 6 |  |
|  | srad03 | 0.912 | 7 |  |
|  | srad02 | 0.912 | 8 |  |
|  | bio4 | 0.911 | 9 |  |
|  | bio15 | 0.911 | 10 |  |
| Dictamnine | tmax03 | 0.916 | 1 |  |
|  | tmean12 | 0.914 | 2 |  |
|  | index_ci | 0.913 | 3 |  |
|  | tmin02 | 0.913 | 4 |  |
|  | bio9 | 0.913 | 5 |  |
|  | bio11 | 0.913 | 6 |  |
|  | tmean1 | 0.912 | 7 |  |
|  | ntl | 0.912 | 8 |  |
|  | bio6 | 0.912 | 9 |  |
|  | tmin12 | 0.912 | 10 |  |
| Obacunone | tmax03 | 0.873 | 1 |  |
|  | bio1 | 0.867 | 2 |  |
|  | bio15 | 0.867 | 3 |  |
|  | tmean4 | 0.865 | 4 |  |
|  | tmax11 | 0.865 | 5 |  |
|  | bio10 | 0.865 | 6 |  |
|  | bio16 | 0.865 | 7 |  |
|  | bio8 | 0.864 | 8 |  |
|  | tmin09 | 0.864 | 9 |  |
|  | index_wi | 0.864 | 10 |  |
| Fraxinellon | tmax01 | 0.875 | 1 |  |
|  | tmin03 | 0.872 | 2 |  |
|  | tmean2 | 0.872 | 3 |  |
|  | tmin02 | 0.872 | 4 |  |
|  | prec4 | 0.872 | 5 |  |
|  | tmin12 | 0.872 | 6 |  |
|  | prec5 | 0.871 | 7 |  |
|  | tmin01 | 0.871 | 8 |  |
|  | bio11 | 0.871 | 9 |  |
|  | bio9 | 0.871 | 10 |  |
| Limonin | prec3 | 0.996 | 1 | Wild |
|  | hsl | 0.996 | 2 |  |
|  | tmin05 | 0.995 | 3 |  |
|  | ntl | 0.995 | 4 |  |
|  | tmean4 | 0.995 | 5 |  |
|  | index_wi | 0.995 | 6 |  |
|  | tmean1 | 0.995 | 7 |  |
|  | tmin06 | 0.995 | 8 |  |
|  | trzdfl | 0.995 | 9 |  |
|  | tmin08 | 0.995 | 10 |  |
| Dictamnine | bio1 | 0.996 | 1 |  |
|  | tmin05 | 0.996 | 2 |  |
|  | tmean10 | 0.996 | 3 |  |
|  | tmax03 | 0.996 | 4 |  |
|  | tmax11 | 0.996 | 5 |  |
|  | tmin09 | 0.995 | 6 |  |
|  | tmean4 | 0.995 | 7 |  |
|  | trylzjhnl | 0.995 | 8 |  |
|  | tmean9 | 0.995 | 9 |  |
|  | ntl | 0.995 | 10 |  |
| Obacunone | tmean12 | 0.995 | 1 |  |
|  | tmin09 | 0.995 | 2 |  |
|  | ntl | 0.995 | 3 |  |
|  | srad11 | 0.995 | 4 |  |
|  | srad10 | 0.995 | 5 |  |
|  | bio1 | 0.995 | 6 |  |
|  | srad06 | 0.995 | 7 |  |
|  | hsl | 0.995 | 8 |  |
|  | srad12 | 0.995 | 9 |  |
|  | srad07 | 0.995 | 10 |  |
| Fraxinellon | prec7 | 0.994 | 1 |  |
|  | tmin09 | 0.994 | 2 |  |
|  | tmean10 | 0.994 | 3 |  |
|  | bio1 | 0.994 | 4 |  |
|  | hsl | 0.993 | 5 |  |
|  | tmean6 | 0.993 | 6 |  |
|  | tmin08 | 0.993 | 7 |  |
|  | tmax03 | 0.993 | 8 |  |
|  | tmax10 | 0.993 | 9 |  |
|  | ntl | 0.993 | 10 |  |

**Table S8.** Results of pearson correlation analysis.

| Environment variables | Cultivation | | | | Wild | | | |
| --- | --- | --- | --- | --- | --- | --- | --- | --- |
|  | Limonin | Dictamnine | Obacunone | Fraxinellon | Limonin | Dictamnine | Obacunone | Fraxinellon |
| bio1 | 0.424* | — | 0.784** | — | — | 0.203 | 0.488* | 0.465* |
| bio10 | — | — | 0.419* | — | — | — | — | — |
| bio11 | — | -0.074 | — | -0.428* | — | — | — | — |
| bio15 | 0.225 | — | 0.631** | — | — | — | — | — |
| bio16 | — | — | -0.401* | — | — | — | — | — |
| bio4 | -0.361 | — | — | — | — | — | — | — |
| bio6 | — | -0.088 | — | — | — | — | — | — |
| bio8 | — | — | 0.403* | — | — | — | — | — |
| bio9 | — | -0.074 | — | -0.448* | — | — | — | — |
| hsl | 0.151 | — | — | — | 0.439* | — | 0.219 | 0.481* |
| index_ci | — | -0.077 | — | — |  | — | — | — |
| index_wi | — | — | 0.519* | — | 0.268 | — | — | — |
| ntl | -0.104 | -0.115 | — | — | 0.440* | 0.070 | 0.329 | 0.522** |
| prec3 | — | — | — | — | -0.747** | — | — | — |
| prec4 | — | — | — | 0.455* | — | — | — | — |
| Prec5 | — | — | — | 0.686** | — | — | — | — |
| prec7 | — | — | — | — | — | — | — | 0.463* |
| srad02 | 0.297 | — | — | — | — | — | — | — |
| srad03 | 0.659** | — | — | — | — | — | — | — |
| srad06 | — | — | — | — | — | — | 0.353 | — |
| srad07 | — | — | — | — | — | — | 0.304 | — |
| srad10 | — | — | — | — | — | — | 0.476* | — |
| srad11 | — | — | — | — | — | — | 0.365 | — |
| srad12 | — | — | — | — | — | — | 0.304 | — |
| tmax01 | — | — | — | -0.301 | — | — | — | — |
| tmax03 | — | -0.067 | 0.634** | — | — | 0.032 | — | 0.181 |
| tmax10 | — | — | — | — | — | — | — | 0.363 |
| tmax11 | — | — | 0.334 | — | — | 0.043 | — | — |
| tmean1 | — | -0.068 | — | — | 0.494* | — | — | — |
| tmean10 | — | — | — | — | — | 0.104 | — | 0.499* |
| tmean12 | — | -0.08 | — | — | — | — | 0.563** | — |
| tmean2 | — | -0.074 | — | -0.453* | — | — | — | — |
| tmean4 | — | — | 0.373 | — | 0.274 | 0.391 | — | — |
| tmean6 | — | — | — | — | — | — | — | 0.482* |
| tmean7 | -0.542** | — | — | — | — | — | — | — |
| tmean9 | — | — | — | — | — | 0.172 | — | — |
| tmin01 | — | — | — | -0.248 | — | — | — | — |
| tmin02 | — | — | — | -0.274 | — | — | — | — |
| tmin03 | -0.604** | — | — | -0.296 | — | — | — | — |
| tmin05 | — | — | — | — | 0.360 | 0.295 | — | — |
| tmin06 | — | — | — | — | 0.307 | — | — | — |
| tmin08 | — | — | — | — | 0.171 | — | — | 0.480* |
| tmin09 | — | — | 0.242 | — | — | 0.260 | 0.444* | 0.495* |
| tmin12 | — | -0.126 | — | -0.184 | — | — | — | — |
| trylzjhnl | — | — | — | — | — | 0.064 | — | — |
| tryxshldj | 0.000 | — | — | — | — | — | — | — |
| trzdfl | — | — | — | — | 0.399 | — | — | — |

Note:*:p＜0.05, **:p＜0.01.

**Table S9.** Factor detection results of main environmental variables of cultivation samples.

| Environment variables | q | *p* |
| --- | --- | --- |
| tmean7 | 0.498 | 0.999 |
| prec5 | 0.719 | 0.049 |
| bio1 | 0.705 | 0.450 |
| bio8 | 0.628 | 0.999 |
| bio10 | 0.628 | 0.999 |
| tmean2 | 0.765 | 0.034 |
| tmax03 | 0.576 | 0.997 |
| srad03 | 0.218 | 0.999 |
| prec4 | 0.272 | 0.999 |
| bio11 | 0.575 | 0.999 |
| bio15 | 0.033 | 0.999 |
| bio9 | 0.575 | 0.999 |
| bio7 | 0.547 | 0.756 |

**Table S10.** Factor detection results of main environmental variables of wild samples.

| Environment variables | q | *p* |
| --- | --- | --- |
| tmean12 | 0.939 | 0.000 |
| tmean6 | 0.316 | 0.985 |
| tmean1 | 0.953 | 0.000 |
| prec3 | 0.451 | 0.291 |
| bio1 | 0.854 | 0.000 |
| prec7 | 0.149 | 0.994 |

**Table S11**. Interaction detection results of main ecological factors of cultivation samples.

|  | tmean7 | prec5 | bio1 | bio8 | bio10 | tmean2 | tmax03 | srad03 | prec4 | bio11 | bio15 | bio9 | bio7 |
| --- | --- | --- | --- | --- | --- | --- | --- | --- | --- | --- | --- | --- | --- |
| tmean7 | 0.498 |  |  |  |  |  |  |  |  |  |  |  |  |
| prec5 | 0.737 | 0.719 |  |  |  |  |  |  |  |  |  |  |  |
| bio1 | 0.783 | 0.847 | 0.705 |  |  |  |  |  |  |  |  |  |  |
| bio8 | 0.648 | 0.769 | 0.788 | 0.628 |  |  |  |  |  |  |  |  |  |
| bio10 | 0.648 | 0.769 | 0.788 | 0.642 | 0.628 |  |  |  |  |  |  |  |  |
| tmean2 | 0.892 | 0.935 | 0.852 | 0.895 | 0.895 | 0.765 |  |  |  |  |  |  |  |
| tmax03 | 0.756 | 0.803 | 0.848 | 0.787 | 0.787 | 0.887 | 0.576 |  |  |  |  |  |  |
| srad03 | 0.664 | 0.896 | 0.904 | 0.785 | 0.785 | 0.89 | 0.739 | 0.218 |  |  |  |  |  |
| prec4 | 0.561 | 0.799 | 0.897 | 0.797 | 0.797 | 0.957 | 0.752 | 0.741 | 0.272 |  |  |  |  |
| bio11 | 0.876 | 0.907 | 0.802 | 0.883 | 0.883 | 0.865 | 0.822 | 0.683 | 0.953 | 0.575 |  |  |  |
| bio15 | 0.679 | 0.908 | 0.892 | 0.823 | 0.823 | 0.918 | 0.740 | 0.313 | 0.365 | 0.812 | 0.033 |  |  |
| bio9 | 0.876 | 0.907 | 0.802 | 0.883 | 0.883 | 0.865 | 0.822 | 0.683 | 0.953 | 0.592 | 0.812 | 0.575 |  |
| bio7 | 0.865 | 0.868 | 0.818 | 0.868 | 0.868 | 0.853 | 0.718 | 0.688 | 0.895 | 0.699 | 0.700 | 0.699 | 0.547 |

**Table S12**. Interaction detection results of main ecological factors of wild samples.

|  | tmean12 | tmean6 | tmean1 | prec3 | bio1 | prec7 |
| --- | --- | --- | --- | --- | --- | --- |
| tmean12 | 0.939 |  |  |  |  |  |
| tmean6 | 0.957 | 0.316 |  |  |  |  |
| tmean1 | 0.957 | 0.986 | 0.953 |  |  |  |
| prec3 | 0.947 | 0.717 | 0.966 | 0.451 |  |  |
| bio1 | 0.97 | 0.878 | 0.981 | 0.946 | 0.854 |  |
| prec7 | 0.952 | 0.617 | 0.967 | 0.781 | 0.94 | 0.149 |
